# The Effect of Gold Nano Particles with Different Sizes on Streptococcus Species

**DOI:** 10.30476/DENTJODS.2021.85219.1119

**Published:** 2021-12

**Authors:** Fatemeh Lavaee, Zahra Ranjbar, Farzan Modaresi, Fatemeh Keshavarz

**Affiliations:** 1 Oral and Dental Disease Research Center, School of Dentistry, Shiraz University of Medical Sciences, Shiraz, Iran; 2 Dept. Oral and Maxillofacial Disease, School of Dentistry, Shiraz University of Medical Sciences, Shiraz, Iran; 3 Dept. of Bacteriology and Virology, Jahrom Medical School, Jahrom University of Medical Sciences, Jahrom, Iran; 4 Undergraduate Students, Student Research Committee, School of Dentistry, Shiraz University of Medical Sciences, Shiraz, Iran

**Keywords:** Nanoparticles, Streptococcus mutans, Streptococcus sanguinis, Streptococcus salivarius

## Abstract

**Statement of the Problem:**

*Streptococcus mutans*, *Streptococcus sanguinis*, and *Streptococcus salivarius* are most common etiologic bacteria for dental caries. Different sizes of gold nanoparticles may have different antibacterial effects on these species.

**Purpose:**

This study aimed to compare the antibacterial effect of chlorhexidine and three sizes of gold nano particles (25, 60, 90nm) against clinical and standard strains of *Streptococcus mutans*, *Streptococcus sanguinis*, and *Streptococcus salivarius*.

**Materials and Method:**

In this cross-sectional study, the specimens were collected from 75 children aged 3-5 years old. Antibacterial effect of chlorhexidine and three sizes of gold nano particles (25, 60, 90nm) were investigated by evaluating the minimum inhibitory concentration (MIC) and minimum bactericidal concentration (MBC) against three bacterial strains.

**Results:**

The MIC and MBC of gold nanoparticles with different sizes against *Streptococcus mutans*, *Streptococcus sanguinis*, and *Streptococcus salivarius* were statistically different. The MIC and MBC of smaller gold nano particles (25nm) were significantly lower (*p*<0.001) than larger ones. Patient-derived bacteria had significantly higher values of MIC and MBC in comparison to standard species (*p*<0.001).

**Conclusion:**

The results of this study confirmed the significant size-dependency of gold nano particles for antibacterial activity. As the size of gold nano particles decrease, the antibacterial properties enhance.

## Introduction

Nano is derived from the Greek word which means ‘dwarf’; nanotechnology is a science that deals with manipulation of matter at the atomic level [ 1 - 2 ].

Nanobiotechnology is a field of applying nano scale techniques and biomaterials for inventing new treatments, medications, drug delivery systems [ 3 - 4 ], enzyme immobilization, and DNA transfection [ 2 ]. This science has been developed greatly. The efficacy of nano-particles can be affected by their size [ 5 - 6 ].

Dental caries is the most common human infectious disease in which diverse pathogenic factors and microorganisms have been identified such as *streptococcus mutans* (*S. mutans*), *streptococcus salivarius* (*S. salivarius*) and *streptococcus sanguinis* (*S. sanguinis*), salivary related disorders and individual diet [ 7 - 8 ].

Different treatments or preventive protocols have been introduced for dental caries. For centuries, metals have been proposed as antibacterial agents. Silver, gold, zinc, platinum [ 4 , 7 ] are the most common metallic agents. The antibacterial properties of metals can be affected by their contact area; larger surface of metals nanoparticles may cause more potent interactions with other molecules, which have not yet been determined [ 7 , 9 ]. Recently gold nanoparticles (AuNPs) have been introduced as a novel platform for new applications including nanobiotechnology and nanobiomedicine. Gold nanoparticles have convenient surface bio conjunction and noticeable Plasmon resonance optional properties. In addition, they have antimicrobial effect and cause bacterial membrane damage, toxicity and aggregation interference [ 10 ]. To the best of our knowledge, there were few studies about comparing different sizes of AuNPs. On the other hand, many evaluations have been confirmed the nanoparticles antibacterial effect. 

Martínez-Castañón *et al*. [ 11 ] evaluated the antibacterial properties of silver nanoparticles (7, 29, 89nm) against *Escherichia coli* (*E.coli*) and *S.aureus*. Decreasing in nanoparticle size, the antibacterial activity increased in the mentioned study. Smaller silver nanoparticles can present greatest surface area, interact with bacteria in a broader surface and reach the nuclear contact more easily. Hernández-Sierra *et al*. [ 7 ] have assessed the effect of silver, zinc oxide and gold nanoparticles with average sizes of 25, 125, 80nm on *S.mutans*. They have confirmed the increase in contact surface by reduction of nanoparticles size. 

A study has reported antibacterial effect of gold and silver nanoparticles against *E.coli* and *Bacillus Calmette-Guerin* [ 5 ]. In addition, this was confirmed for silver and AuNPs against *E.coli* and *S.aureus* [ 3 ]. According to these researches, we aimed to evaluate antibacterial effects of different sizes of AuNPs against dental biofilm bacteria (such as *S.mutans*, *S.salivarius* and *S.sanguinis*).

## Materials and Method

In this study, 75 children aged 3-5 years old, referred to Shiraz Dental Faculty, were enrolled in this study during 6 months. The Ethics Committee of Shiraz University of Medical Sciences has been approved this study (IR.SUMS.REC.1395.S1017). This study has been conducted according to the *Declaration of Helsinki* (1975). One of the participant’s parents signed the written consent form. Dental caries of children was assessed by using dental explorer and bitewing radiographs [ 12 ]. A total of 75 specimens from teeth plaque with dental caries were achieved by a sterile toothpick. In addition, a sterile cotton swab was employed for collecting unstimulated saliva from sublingual region. The samples were inserted into separate 1.0-mL reduced transport fluid vials [ 13 ] and sent to the microbiologic center (located in Jahrom, Fars province, Iran) for processing and laboratory evaluations. The saliva and plaque samples were diluted and placed on MM10-sucrose agar [ 14 ].

The cultures were incubated anaerobically (85% N_2_, 10% CO_2_, and 5% H_2_) and *S. sanguinis* colonies were selected based on their firm, adherent, star-shaped colony morphology [ 15 - 16 ], also those colonies with spherical and gram-positive anaerobic bacteria that were catalase and oxidase negative were *S.salivarius*. 

These discrete colonies were placed on proper medium in order to detect the hydrolysis of arginine and lack of mannitol fermentation in order to differentiate *S.mutans* from *S. sanguinis*. 

The prototype strain of *S. sanguinis* (ATCC 10556), *S. mutans* (ATCC 25175) and *S.salivarius* (ATCC9759) were used as standard species.

**Figure 1:**
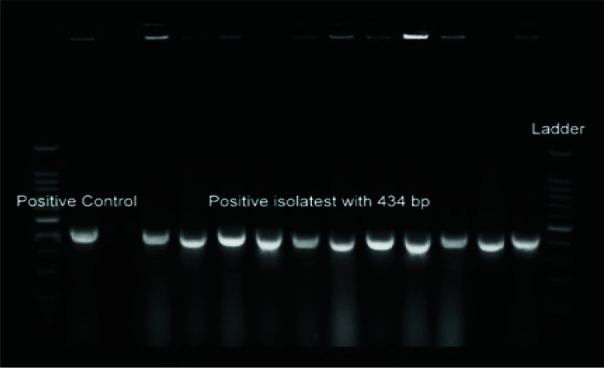
PCR amplification of patient-isolated *S. mutans* species in this study. The electrophoresis agarose gel was stained with 0.5 µg/ml ethidium bromide and the figure was prepared by UV gel documentation system. Positive control *S. mutans* (ATCC 25175) (433bp) is also seen in this figure

**Figure 2:**
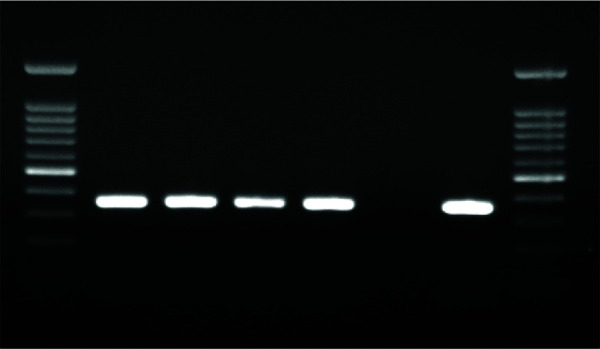
PCR amplification of patient-isolated *S. sanguinis* species in this study. The electrophoresis agarose gel was stained with 0.5 µg/ml ethidium bromide and the figure was prepared by UV gel documentation system. Positive control (313bp) *S.sanguinis* (ATCC 10556) is also seen in this figure

**Figure 3:**
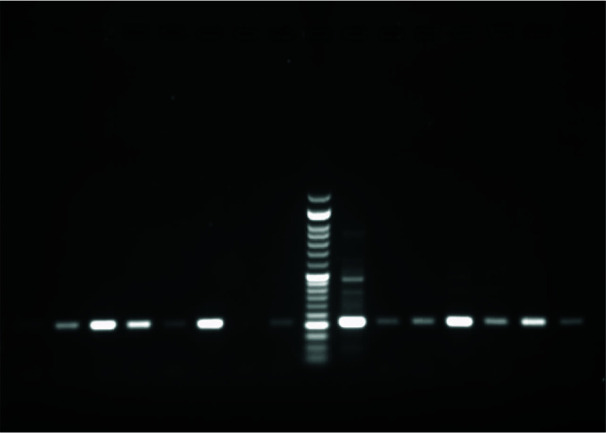
PCR amplification of patient-isolated *S. salivarius* species in this study. The electrophoresis agarose gel was stained with 0.5 µg/ml ethidium bromide and the figure was prepared by UV gel documentation system. Positive control *S. salivarius* (ATCC9759) (544bp) is also seen in this figure

Polymerase chain reaction (PCR) was used for biochemical tests confirmation of all obtained specimens and detection of the *S. mutans*, *S. sanguinis* and *S. salivarius* by primers pairs [ 17 ]. These primers were 5-GqaG-CACCACAACATTGGGAAGCTCAGTT and 5-GGAATG-GCCGCTAAGTCAACAGGAT for S. mutans (433bp) and GGATAGTGGCTCAGGGCAGCCAGTT and GAACAGT-TGCTGGACTTGCTTGTC for *S. sanguinis* and MKK-GTGTTGCCACATCACTCGCTTCGG and MKK-CGTTG-ATGTGCTTGAAAGGGCACCATT for *S. salivarius* (544 bp). The amplicons were 433bp, 313bp and 544bp size (respectively Figures 1, 2 and 3). 

Blast analysis was used for assessing the candidate primers sequences in the database (http://www.ncbi. nlm.nih.gov/GenBank). 

The genomic DNA was extracted according to the manufacture direction (kit: Thermo Science, Vilnius, Lithuania). The genomic DNA was extracted according to the manufacture direction (kit: Thermo Science, Vilnius, Lithuania). PCR was performed in gradient thermal cycler (Biometra-T Gradient, Whatman Biometra, Göttingen, Germany). The Annealing temperature was confirmed by gradient system. PCR amplification of patients isolated S.mutans, S. sanguinis and S. salivarius species in this study are shown in Figure 1, 2 and 3. The electrophoresis agarose gel was stained with 0.5 µg/ml ethidium bromide and the figure was prepared by UV gel documentation system.

 The antibacterial assessment was conducted on clinical isolated bacteria (*S. mutans*, *S. sanguinis* and *S. salivarius*) and standard species including *S. mutans* (ATCC 25175), *S. sanguinis* (ATCC 10556) and *S. Salivarius* (ATCC 9759) from the Pasteur Institute, Tehran, Iran.

 They were sub-cultured in 5% sheep’s blood agar. At first, five to six colonies from an overnight culture were diluted in brain heart infusion broth and were incubated in an aerobic environmental condition for 1-2 hours at 35°C to reach the concentration of 1.5×10^8^ CFU/ml. The final colonies concentration of 1.5×10^6^ CFU/m were achieved by saline solution. 

**Figure 4:**
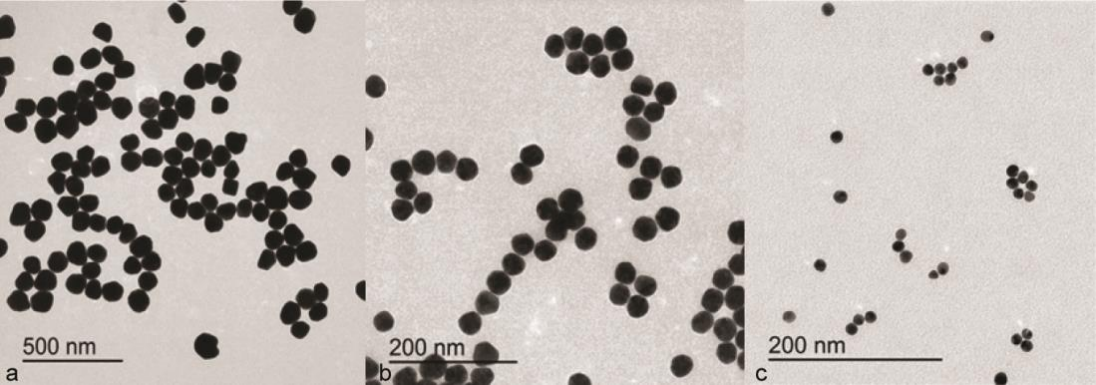
Gold nano particles with size of 90, 60, and 25nm in a, b, c respectively

The AuNPs with different sizes including 25, 60, and 90nm were selected for this study (Biometra-T Gradient, Whatman Biometra, Göttingen, Germany). According to the supplier, nanoparticles were more than 99% pure after ignition. A water- based solution of nanoparticles was prepared. The nanoparticles size distribution was confirmed by ultraviolet-visible spectroscopy (Shimatzu, Kyoto, Japan) and a particle size analyzer (Zetasizer, Nano-ZS, Malvern, Herrenberg, Germany) (Figure 4). Mean size ranged from 25 to 90nm for nanoparticles.

Colloidal solutions of nanoparticles with initial concentration of 500µg/ml, were sterilized in gravity autoclave before anti-microbial tests.

The minimum inhibitory concentration (MIC) and minimum bactericidal concentration (MBC) were assessed in this study. The MICs for prepared solutions were assessed by spectrophotometric microdilution method (SMM) and turbidity. For each strain, we used a 96-well ELISA plate, and solutions were colored with resazurin (Sigma, St Louis, MO, USA). Row 1 was filled with chlorhexidine as control and 140µL BHI (brain heart infusion agar), 50µl of control solution and 10µl of bacterial culture (about 108 colony-forming units/mL). Pure culture media and bacterial solution were poured in a row as negative and positive control.

Three rows were filled with 100µl of experimental solutions, 100µl BHI and 10µl of culture. Then they were incubated at 37°C for 18 hours, and before and after incubation, automatic ELISA tray reader (Readwell Plate) adjusted at 524 nm specified the absorbance of each well, then to assure the true viability of antimicrobial activity, all wells were filled with the oxidation-reduction indicator resazurin [ 18 ].

A row for checking the viability of bacteria strains, and another one for assessing the sterility of experimental solutions, and the medium were considered and nanoparticles alone were added in another row. 

MBC was specified when no visible bacterial growth on plates with Brain Heart Infusion Agar which had been incubated at 37°C for 24 hours was detected. All tests were conducted three times.

 Evaluation the MIC and MBC values of AuNPs different sizes against standard species of *S.mutans*, *S.sanguinis* and *S.salivarius* was also performed, data were analyzed by SPSS version 18. In order to compare the MIC and MBC values two way ANOVA and Poshoc Tukey were used.

## Results

The MIC and MBC of AuNPs against patient-isolated bacteria and standard species of *S.mutans*, *S.salivarius*, and *S.sanguinis* are reported in Table1 and 2 respectively. The MIC and MBC of different sizes (25, 60, 90nm) of AuNPs against different patient-isolated bacteria and standard species (*S.mutans*, *S.salivarius* and *S. sanguinis*) were statistically different (*p*< 0.001). The only exceptions are the MIC and MBC of 60 and 90nm AuNPs against *S.mutans* and *S.salivarius*, which were not significantly different. For patient-isolated bacteria and standard spices, the reported MIC and MBC of 25 nm AuNPs against all three spices of streptococcus (*S.mutans*, *S.salivarius* and *S.sanguinis*) were very lower than 60 and 90nm AuNPs. In addition, *S.mutans* was more susceptible for AuNPs than *S.salivarius* and *S.sanguinis*. For instance, 25nm AuNPs had inhibitory effect at minimum concentration of 1.73±1.23µg/ml against S.mutans, while MIC was 91.61±46.39µg/ml for 60nm and 232.95±124.53µg/ml for 90nm AuNPs (Table 1).

**Table 1:** Minimum inhibitory and minimum bactericidal concentration mean of gold nanoparticles against patient- derived *S.mutans*, *S.salivarius*, *S.sanguinis*

Size (nm) bacteria		MIC mean(µg/ml)	MBC mean(µg/ml)
25	*S.mutans*	1.73±1.23	4.05±2.68
	*S.sanguinis*	3.17±1.50	6.46±2.98
	*S.salivarius*	2.86±1.58	6.09±3.15
	Total	2.51±1.55	5.41±3.09
60	*S.mutans*	91.61±46.39	184.65±91.37
	*S.sanguinis*	148.21±64.46	289.28±124.15
	*S.salivarius*	119.31±63.20	242.42±124.76
	Total	117.46±61.82	234.37±119.97
90	*S.mutans*	232.95±124.53	217.26±236.59
	*S.sanguinis*	353.57±130.34	329.53±326.91
	*S.salivarius*	329.54±124.28	302.53±314.42
	Total	299.10±136.42	277.47±294.03

**Table 2:** Minimum inhibitory and minimum bactericidal concentration mean of gold nanoparticles against standard *S.mutans*, *S.salivarius*, *S.sanguinis*

AuNPs	*S. salivarius* (ATCC 9759)		*S. sanguinis* (ATCC10556)		*S. mutans* (ATCC 25175)	
	MBC (µg/ml)	MIC (µg/ml)	MBC (µg/ml)	MIC (µg/ml)	MBC (µg/ml)	MIC (µg/ml)
25nm	1.95	0.97	3.9	1.95	7.81	1.95
60nm	125	62.5	125	62.5	250	125
90nm	500	250	1000	500	500	250
Chlorhexidine	50	50	50	25	50	50

The patient-isolated bacteria had higher level of MIC and MBC for AuNPs in comparison to standard species of streptococcus.

**Figure 5:**
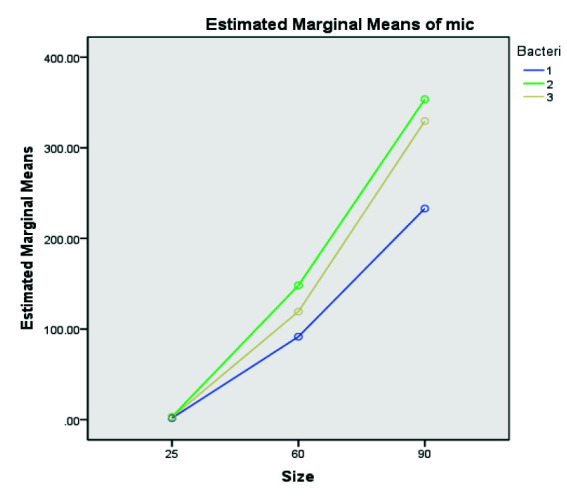
The minimum inhibitory concentration (MIC) value of AuNPs against S.mutans (1), S.sanguinis, (2) and S. salivarius (3)

**Figure 6:**
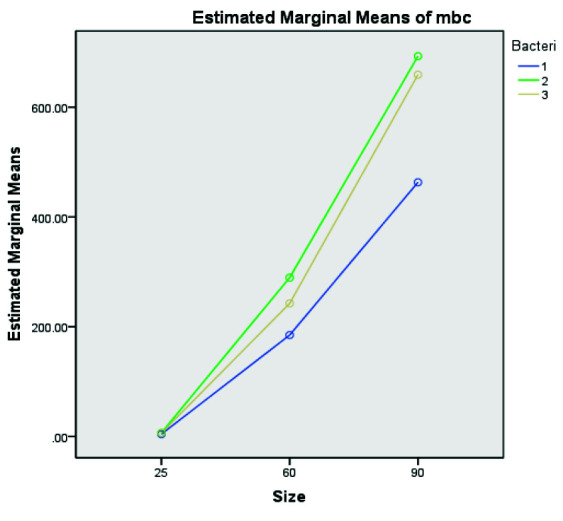
The minimum bactericidal concentration (MBC) value of AuNPs against *S.mutans* (1), *S.sanguinis*, (2) and *S.salivarius* (3)

The MIC and MBC of chlorhexidine against all three patient-isolated and standard spices of streptococcus (Table 2), which have been evaluated in this study, were very higher than values that have been registered for 25nm AuNPs. In addition, chlorhexidine affected all three evaluated species of bacteria similarly. The MIC and MBC values of AuNPs against patient-isolated *S.mutans*, *S.sanguinis*, and *S.salivarius* are represented in Figure 5 and 6 respectively.

## Discussion

The antibacterial properties of AuNPs were inversely size dependent. The smallest AuNPs (25nm) was the most potent antibacterial agent (*p*< 0.001) and the lowest MIC and MBC has been reported for 25nm AuNPs against evaluated both patient-isolated and standard bacteria. The 25nm AuNP was more potent than chlorhexidine against evaluated standard species of *S.mutans*, *S.salivarius* and *S.sanguinis*. The registered MIC and MBC against patient-isolated bacteria were higher than values of standard species.

The antibacterial activity of nanoparticles has been investigated in many studies in which different nanoparticles ions showed diverse range of antibacterial activity against different bacteria, gram positive and gram negative [ 5 - 6 , 11 ].

Agnihotri *et al*. [ 6 ] confirmed size-specific antibacterial efficacy of silver nanoparticles against *Escherichia coli* (*E.coli*) and *Staphylococcus aureus* (*S.aureus*). Silver nanoparticles smaller than 10 nm showed considerable enhancement in antimicrobial activity; moreover, the smallest size mediated the fast bactericidal activity. 

Findings of another study about the effect of size and shape of silver nanoparticles were in consistent with previous researches. MIC of 7nm silver nanoparticles against *S. aureus* and *E.coli* were the lowest [ 2 ].

In accordance to these studies Zhou *et al*. [ 5 ] evaluated silver nanoparticles antimicrobial effect against both aerobic and anaerobic oral pathogen; confirming the size-dependency of antibacterial activity of nanoparticles, the reported MIC for 5 and 15nm silver nanoparticles against *S.mutans* and *S.sanguinis* were 50µg/ml.

Hernández-Sierra *et al*. [ 7 ] reported MIC of 4.86µg/ ml for 25nm silver nanoparticles against *S.mutan*. 

These values are different from our reports for AuNPs against *S.mutans* (MIC 25nm AuNPs=1.73µg/ ml) and *S.sanguinis* (MIC 25nm AuNPs=3.17 µg/ml). 

These differences may be related to different concentration and type of nanoparticle in addition to the method of measuring MIC. 

A study in 2009 has reported the higher susceptibility of standard *S.mutans* to silver nanoparticles in comparison to the clinical isolated strains [ 19 ]. This is in line with findings of Yamamoto *et al*. [ 20 ] and presenting study. However, for 90nm AuNPs there were some violated case. Although there are more reports about antibacterial effect of silver nanoparticles [ 7 , 21 ], few studies have evaluated and confirmed antibacterial activity of other kinds of nanoparticles such as Zinc Oxide nanoparticles [ 20 , 22 - 23 ], Zn nanoparticles, and AuNPs [ 7 ]. Raghupathi *et al*. [ 22 ] have confirmed size dependency of ZnO nanoparticles with bacterial growth inhibitory power. In spite of using different nanoparticles, the results of previous studies are consistent with our findings. Moreover, different size and concentration of nanoparticles can play an important role in determination of the inhibitory effect on microorganisms. Although the precise antibacterial mechanism of nanoparticles is unclear, there are some theories, which explain their mechanism [ 24 - 25 ]. Nanoparticles can attach to the cell membrane and disturb the permeability of the outer membrane. Therefore, they can enter the inner layer of membrane and stop respiratory chain dehydrogenase [ 24 - 25 ], disassociate the respiratory chain and oxidative phosphorylation and disable proton- motive force via cytoplasmic membrane [ 26 ]. Diminishing the size of nanoparticles can lead to more surface area of interaction whit bacterial cell membrane and increasing gold ions release and better antibacterial properties [ 19 ].

Electrostatic attraction between bacterial cell membrane and nanoparticles, produce a tendency to enhance nanoparticles accumulation [ 27 - 28 ] on bacterial cell membrane, which can lead to high stress in bacterial membrane and penetration of nanoparticles to cytoplasm and finally cell lysis [ 5 ]. In theory, interaction of nanoparticles with thiol groups of bacterial proteins may affect the DNA replication [ 29 ]. 

Several researches have theorized two possible mechanisms of antibacterial activity including increase in reactive oxygen species (ROS) production (hydroxyl radicals and singlet oxygen) [ 30 - 31 ] and disruption of cellular function by accumulation of nanoparticles on bacterial cell wall, in the cytoplasm or periplasm region [ 32 - 33 ]. Some nanoparticles can affect the bacterial junction and expression of cytokine gene [ 34 ].

The effect of nanoparticles on bacterial respiration can be explained by more resistancy of anaerobic oral bacteria such as *S.mutans*, *S.sanguinis*, *S.mitis* and *Actinobacillus actinomycetemcomitans*. For anaerobic bacteria, the release of nanoparticles may be blocked by insufficient air; hence, the difference in releasing ions of nanoparticles makes the diversities in antimicrobial potencies for aerobic and anaerobic bacteria [ 35 ].

Beside this item, the effect of nanoparticles on gram-negative and gram-positive species is different because of different width of their cellular wall [ 36 ].

Although there is a controversy about the relation of concentration of nanoparticles and antibacterial effect [ 5 , 35 ], all articles support the size - dependency in a similar manner; as the size of nanoparticles decrease, the antimicrobial effect increase [ 7 , 11 , 19 , 35 ].

In the current study, the antibacterial potency of three different sizes of AuNPs was evaluated on both clinically isolated and standard species. This can help evaluate the trend of resistancy in oral pathogens.

According to findings of this study, unfortunately there is an increasing and concerning trend of antimicrobial resistancy in human isolated microorganisms. Therefore, the need for introducing new antimicrobial agents is completely necessary. 

AuNP has been selected in our study for evaluation because of its solubility in water and in culture media. Using water as our solvent can eliminate the antibacterial effects of other kinds of solvents as a confounding factor. Reviewing the literature revealed that more studies have used AuNPs with larger size than presenting study. The method of preparing nanoparticles and their concentration for determination of AuNPs antibacterial efficacy was different. There is a concentration limitation for nanoparticles in order to show their best antimicrobial properties. More concentration may lead to nanoparticles agglomeration and diminishing their size dependent properties.

In this study, the small sizes of AuNP have been used for antibacterial assessment. Future belongs to new commercial nano containing antimicrobial and antibiofilm agents. Further *in vivo* evaluations for new designed nano products such as dentifrices and mouthwashes can be recommended for future studies.

## Conclusion

The results of this study confirmed the significant size- dependency of AuNPs for antibacterial activity. As the size of AuNPs decrease, the antibacterial properties enhance. The patient-isolated bacteria are more resistant to antibacterial effect of AuNPs.

## Conflict of Interest

The authors declare that they have no conflict of interest.

## References

[1] Abiodun-Solanke IMF, Ajayi DM, Arigbede AO ( 2014). Nanotechnology and its Application in Dentistry. Ann Med Health Sci Res.

[2] Ozak ST, Ozkan P ( 2013). Nanotechnology and dentistry. European J Gen Dent.

[3] MubarakAli D, Thajuddin N, Jeganathan K, Gunasekaran M ( 2011). Plant extract mediated synthesis of silver and gold nanoparticles and its antibacterial activity against clinically isolated pathogens. Colloids Surf B Biointerfaces.

[4] Annamalai A, Christina VLP, Sudha D, Kalpana M, Lakshmi PTV ( 2013). Green synthesis, characterization and antimi-crobial activity of Au NPs using Euphorbia hirta L. leaf extract. Colloids Surf B Biointerfaces.

[5] Hernández-Sierra JF, Ruiz F, Pena DCC, Martínez-Guti-érrez F, Martínez AE, Guillén AdJP, et al (2008). The antimicrobial sensitivity of Streptococcus mutans to nanoparticles of silver, zinc oxide and gold. Nanomedicine.

[6] Ghapanchi J, Moattari A, Lavaee F, Shakib M ( 2015). The antibacterial effect of four mouthwashes against Streptococcus mutans and Escherichia coli. JPMA.

[7] Lavaee F, Faez K, Hadi N, Modaresi F ( 2016). Antimicrobial and antibiofilm activity of silver, titanium dioxide and iron nano particles. Am J Dent.

[8] Ghanavati Behbahan F, Salari M, Mousavi SR, Rezaei R ( 2016). Antimicrobial activities of Gold nanoparticles against Sa-lmonella typhimurium. Advanced Herb Med.

[9] Martínez-Castañón GA, Niño-Martínez N, Martínez-Gutierrez F, Martínez-Mendoza JR, Ruiz F ( 2008). Synthesis and antibacterial activity of silver nanoparticles with different sizes. J Nanopart Res.

[10] Zhou Y, Kong Y, Kundu S, Cirillo JD, Liang H ( 2012). Antibacterial activities of gold and silver nanoparticles against Escherichia coli and bacillus Calmette-Guerin. J Nanobiotechnology.

[11] Wenzel A ( 1998). Digital radiography and caries diagnosis. Dentomaxillofac Radiol.

[12] Caufield PW, Dasanayake AP, Li Y, Pan Y, Hsu J, Hardin JM ( 2000). Natural History of Streptococcus sanguinis in the Oral Cavity of Infants: Evidence for a Discrete Window of Infectivity. Infection immunity.

[13] Loesche WJ, Rowan J, Straffon LH, Loos PJ ( 1975). Association of Streptococcus mutants with human dental decay. Infection Immunity.

[14] Marsh P, Featherstone A, McKee A, Hallsworth A, Robinson C, Weatherell J, et al ( 1989). A microbiological study of early caries of approximal surfaces in schoolchildren. J Dent Res.

[15] Jordan HV, Laraway R, Snirch R, Marmel M ( 1987). A simplified diagnostic system for cultural detection and enumeration of Streptococcus mutans. J Dent Res.

[16] Garnier F, Gerbaud G, Courvalin P, Galimand M ( 1997). Identification of clinically relevant viridans group streptococci to the species level by PCR. J Clin Microbiol.

[17] Najjar MB, Kashtanov D, Chikindas ML ( 2009). Natural antimicrobials ε-poly-l-lysine and Nisin A for control of oral microflora. Probiotics Antimicrobial Proteins.

[18] Agnihotri S, Mukherji S, Mukherji S ( 2014). Size-controlled silver nanoparticles synthesized over the range 5–100 nm using the same protocol and their antibacterial efficacy. RSC Advances.

[19] Espinosa-Cristóbal L, Martínez-Castañón G, Martínez-Martínez R, Loyola-Rodriguez J, Patino-Marin N, Reyes-Macias J, et al ( 2009). Antibacterial effect of silver nanoparticles against Streptococcus mutans. Mater Lett.

[20] Yamamoto O ( 2001). Influence of particle size on the antibacterial activity of zinc oxide. Solid State Sci.

[21] Pal S, Tak YK, Song JM ( 2007). Does the Antibacterial Activity of Silver Nanoparticles Depend on the Shape of the Nanoparticle? A Study of the Gram-Negative Bacterium Escherichia coli. Appl Environ Microbiol.

[22] Raghupathi KR, Koodali RT, Manna AC ( 2011). Size-dependent bacterial growth inhibition and mechanism of antibacterial activity of zinc oxide nanoparticles. Langmuir.

[23] Nagarajan P, Rajagopalan V ( 2008). Enhanced bioactivity of ZnO nanoparticles—an antimicrobial study. Sci Technol Adv Mater.

[24] Rai M, Yadav A, Gade A ( 2009). Silver nanoparticles as a new generation of antimicrobials. Biotechnology advances.

[25] Lok CN, Ho CM, Chen R, He QY, Yu WY, Sun H, et al ( 2006). Proteomic analysis of the mode of antibacterial action of silver nanoparticles. J Proteome Res.

[26] Holt KB, Bard AJ ( 2005). Interaction of silver(I) ions with the respiratory chain of Escherichia coli: an electrochemical and scanning electrochemical microscopy study of the antimicrobial mechanism of micromolar Ag+. Biochemistry.

[27] Eby DM, Luckarift HR, Johnson GR ( 2009). Hybrid antimicrobial enzyme and silver nanoparticle coatings for medical instruments. ACS Appl Mater Interfaces.

[28] Goodman CM, McCusker CD, Yilmaz T, Rotello VM ( 2004). Toxicity of gold nanoparticles functionalized with cationic and anionic side chains. Bioconjug Chem.

[29] Marini M, De Niederhausern S, Iseppi R, Bondi M, Sabia C, Toselli M, et al ( 2007). Antibacterial activity of plastics coated with silver-doped organic-inorganic hybrid coatings prepared by sol-gel processes. Biomacromolecules.

[30] Yang H, Liu C, Yang D, Zhang H, Xi Z ( 2009). Comparative study of cytotoxicity, oxidative stress and genotoxicity induced by four typical nanomaterials: the role of particle size, shape and composition. J Appl Toxicol.

[31] Xia T, Kovochich M, Liong M, Mädler L, Gilbert B, Shi H, et al ( 2008). Comparison of the Mechanism of Toxicity of Zinc Oxide and Cerium Oxide Nanoparticles Based on Dissolution and Oxidative Stress Properties. ACS Nano.

[32] Zhang L, Jiang Y, Ding Y, Povey M, York D ( 2007). Investigation into the antibacterial behaviour of suspensions of ZnO nanoparticles (ZnO nanofluids). J Nanopart Res.

[33] Brayner R, FerrariIliou R, Brivois N, Djediat S, Benedetti MF, Fievet F ( 2006). Toxicological impact studies based on Escherichia coli bacteria in ultrafine ZnO nanoparticles colloidal medium. Nano Lett.

[34] Roselli M, Finamore A, Garaguso I, Britti MS, Mengheri E ( 2003). Zinc oxide protects cultured enterocytes from the damage induced by Escherichia coli. J Nutr.

[35] Lu Z, Rong K, Li J, Yang H, Chen R ( 2013). Size-dependent antibacterial activities of silver nanoparticles against oral anaerobic pathogenic bacteria. J Mater Sci Mater Med.

[36] Thiel J, Pakstis L, Buzby S, Raffi M, Ni C, Pochan D, et al ( 2007). Antibacterial properties of silver-doped Titania. Small.

